# Musculoskeletal pain is prevalent in Chinese medical and dental students: A cross-sectional study

**DOI:** 10.3389/fpubh.2022.1046466

**Published:** 2022-11-24

**Authors:** Yunzhi Lin, Xuehui Zhang, Hongyan Li, Ying Huang, Wenming Zhang, Chaofan Zhang

**Affiliations:** ^1^Department of Stomatology, The First Affiliated Hospital, Fujian Medical University, Fuzhou, China; ^2^Department of Stomatology, National Regional Medical Center, Binhai Campus of the First Affiliated Hospital, Fujian Medical University, Fuzhou, China; ^3^School of Health Management, Fujian Medical University, Fuzhou, China; ^4^Department of Orthopaedic Surgery, The First Affiliated Hospital, Fujian Medical University, Fuzhou, China; ^5^Department of Orthopaedic Surgery, National Regional Medical Center, Binhai Campus of the First Affiliated Hospital, Fujian Medical University, Fuzhou, China; ^6^Fujian Provincial Institute of Orthopedics, The First Affiliated Hospital, Fujian Medical University, Fuzhou, China

**Keywords:** musculoskeletal pain (MSP), medical students, dental students, survey, questionnaire

## Abstract

**Objectives:**

Musculoskeletal pain (MSP) is a major reason for consultation in primary care and is becoming increasingly prevalent among medical students. There is little research on the current situation of MSP among Chinese medical and dental students. Data on the analysis of risk factors related to MSP are also limited. The objectives of this study were to investigate the prevalence and characteristics of MSP among Chinese medical and dental students and to explore the risk factors for MSP and students' intent to seek medical treatment.

**Methods:**

An anonymous, internet-based, cross-sectional, open survey was distributed to medical and dental students at Fujian Medical University, Fuzhou, China. Data on the demographic information and characteristics of MSP were collected and analyzed. In addition to descriptive statistics, logistic regression was used to analyze significant risk factors contributing to MSP.

**Results:**

A total of 1,178 students responded to the survey (response rate = 79.6%), including 722 medical students and 456 dental students. The age ranged from 16 to 24. There were 553 male students and 625 female students. A total of 523 students reported neck pain (NP, 44.4%), 361 students reported low back pain (LBP, 30.6%), and 182 students reported joint pain (JP, 15.4%). Sixty-six students (5.6%) simultaneously suffered from NP, LBP, and JP. The prevalence of NP (49.1 vs. 41.4%, *P* = 0.01), LBP (34.6 vs. 28.1%, *P* = 0.02), and JP (20.2 vs. 12.5%, *P* < 0.001) was significantly higher in dental students than in medical students. The prevalence of MSP was significantly different among the academic years for NP and LBP (*P* = 0.02 and *P* < 0.001, respectively). Univariate and multivariate regression analyses demonstrated that female sex, PSS-10 score, and major of stomatology were risk factors for MSP. Medical and dental students' intention to seek treatment for MSP was low and was significantly associated with the severity of pain.

**Conclusions:**

The prevalence of MSP in Chinese medical and dental students is high, especially for NP and LBP, and is significantly higher in dental students than in medical students. The prevalence of NP and LBP were significantly different among academic grades. Female sex, PSS-10 score, and major of stomatology were risk factors for MSP. Students' intent to seek treatment for MSP was very low and was determined by the severity of pain.

## Introduction

Pain is a major clinical, social, and economic problem in communities all around the globe (1). A study estimated that approximately half of the people have experienced an episode of pain that lasted at least 1 day (2). Musculoskeletal pain (MSP), which is a symptom of various types of pain (neck pain, limb pain, low back pain, joint pain, chronic widespread pain), is a major reason for consultation in primary care (3). The burden of MSP is increasing because of the rapid aging of populations, especially in developing countries, such as China (4).

MSP induces discomfort in patients and also has a large impact on many other aspects of older people's health, such as low physical activity level, poor mobility, frailty, depression, cognitive impairment, falls, and poor sleep quality (4). MSP is common among university students and increases year by year. A cross-sectional survey of Finnish university students found that the prevalence of MSP significantly increased from 2000 to 2012, with the prevalence rate of neck-shoulder pain increasing from 25 to 29%, lower back pain increasing from 10 to 14%, and limb and joint pain increasing from 7 to 8% (5). The prevalence of MSP seems to be even higher in medical students (6, 7), as students have to undergo long-term medical training, during which students are exposed to stress, study problems, and long training hours in hospital wards and clinics (8). The goals of a medical school are to produce competent, professional doctors and promote the health care of society. However, during the period of medical training, students are exposed to stress, study problems, and long training hours in hospital wards and clinics (8). In addition to the increasing use of computers in teaching and learning, MSP has become even more prevalent among medical students (9). One comparative study demonstrated that MSP was significantly more prevalent in medical students than in non-medical students (10). MSP affects the quality of productivity and absenteeism from university lessons (11) and also causes mental issues, which affect students' future careers (12). Data has demonstrated that musculoskeletal problems have become the most commonly reported complaint among healthcare professionals (10).

Several studies have evaluated MSP problems in medical and dental students around the globe, all reporting a high prevalence in such populations (7, 13, 14). However, there is still little research on the current situation of MSP among Chinese medical students. Chinese medical students face huge pressure during school, an extremely long length of schooling, and also suffer from severe mental diseases, particularly depression and anxiety, which might be caused by worries about horrible working conditions, such as the stressful relationship between patients and doctors (15). It was estimated that the average prevalence of depression and anxiety among Chinese medical students was 32.74 and 27.22%, respectively (15). In addition, there is a lack of comparative analysis between medical students and dental students. Due to the different nature of work, the prevalence and characteristics of MSP in these two groups are thought to be different. The literature has shown a seemingly higher prevalence of MSP in dental students than in medical students (6, 7, 13, 14), but to simply compare the value would be unscientific and introduce bias. To explore the characteristics of MSP in these two groups of students would not only provide valuable information for the students but also provide useful information for the specific curriculum designers, such as setting up relevant courses to popularize the knowledge of MSP. Furthermore, most of the literature lacks an analysis of risk factors related to MSP in medical and dental students, and it is unclear what factors can contribute to MSP in medical and dental students.

Therefore, this study aimed to investigate the prevalence and characteristics of MSP among medical and dental students in a large medical university in China. Specifically, we aimed to explore the differences in MSP characteristics between medical students and dental students. Second, this study aimed to explore the risk factors that affect the prevalence of MSP in medical and dental students, as well as students' intention to seek medical treatment.

## Methods

### Study participants and design

This study was approved by the Ethics Committee of our institution [2022[341]]. The sample was recruited from undergraduate medical and dental students at Fujian Medical University, Fuzhou, China, which is the largest medical university in Fujian Province in southeast China. The study adopted a cross-sectional design using a web-based survey. The survey was anonymous and self-administered online, with a link to the questionnaire sent to all registered medical and dental students. WeChat (the most popular social media platform in China) was used to send out the survey, and it was sent only once. In total, 900 medical students from the discipline of clinical medicine (bachelor's and master's degree combined program) in academic years 1–5 (180 in each year) and 579 students from the discipline of dentistry (120, 121, 125, 114, and 99 in academic years 1, 2, 3, 4, and 5, respectively) at the university received the invitation to participate in the survey. The sample size was calculated based on a response rate of 50%, confidence interval of 99%, margin error of 5%, and a total population of 1,479 students. The total sample size required for this study was 918. Students were informed that their participation was voluntary. Online informed consent was obtained from each study participant. Students who met the following conditions were excluded from the study: (1) History of a mental or psychological problem; (2) History of hypertension, diabetes, or other congenital diseases; (3) body mass index (BMI) over 30.0.

### Measures

The questionnaire mainly includes five sections: (1) demographic information (including age, sex, height, weight, ethnicity, history of hypertension, diabetes, or other congenital diseases, drinking history and daily consumption of alcohol, smoking history and daily consumption of cigarettes, mother's education level, father's education level, family annual income, and personal monthly income) and (2) the time spent on five dimensions of daily activities, including exercising, sleeping, studying, sitting and walking (categorized as <1, 1–3, 3–5, 5–7, and >7 h per day), which is used to assess the lifestyle of students (7). The time spent on computers and mobile phones was also asked. (3) The prevalence and characteristics of MSP include neck pain (NP), low back pain (LBP), and joint pain (JP). For the participants with pain, further investigation was made on the history of trauma in the associated region, the severity of pain, the frequency of pain, and the intention of treatment. The numeric rating scale (NRS) was used to measure the severity of pain, from a scale of 0 for “no pain” to 10 for “most severe pain.” The response options for pain frequency were “Almost Never, Sometimes, Fairly Often, and Very Often.” The response options for treatment were “Seeking medical treatment (Consult a doctor),” and “Conservative treatment (rest, wearing braces, or protective equipment, or taking analgesic drugs).” (4) The Perceived Stress Scale (PSS)-10 questionnaire. The PSS-10 score was used to evaluate the stress of students. The PSS-10 is a 10-item questionnaire that assesses the degree of unpredictability and uncontrollability of situations in the participant's life (16). The sum of scores for all 10 questions gives a PSS score for overall perceived stress, with a higher score indicating more stress. The PSS questionnaire is widely employed and has strong normative data and strong reliability and validity (17). (5) The Oswestry Disability Index (ODI) questionnaire. A modified ODI questionnaire combining standardized questions from the neck, low back, and joint ODI questionnaires was used in this study. The ODI questionnaire assesses the degree of severity of 15 quality-of-life issues that students associate with NP, LBP, and JP. The sum of degrees of severity of all 15 questions gives an ODI score for overall quality of life issues due to MSP, with a higher score indicating the more severe quality of life issues. Common questions found in the neck, low back, and joint ODI surveys were combined (7).

The English version of the questionnaire is shown in Appendix. The questionnaire was content-validated by several panel experts in the field of clinical medicine, stomatology, medical education, and health researchers to ensure the relevance and clarity of the questions. A draft questionnaire was pilot tested for comprehension and ease of completion with a group of 20 students not included in the final study population. The questionnaire was then tested for reliability and validity. Section A was on demographic information. For section B, tests showed a reliability (Cronbach's alpha) of 0.759 and validity (Kaiser-Meyer-Olkin test coefficient, KMO) of 0.668. For section C, a Cronbach's alpha of 0.750 and KMO of 0.681 were achieved. For section D, a Cronbach's alpha of 0.807 and KMO of 0.789 was achieved. This data indicates that the questionnaire has good reliability and validity. After completion, surveys were collected, and responses were manually entered into a password-protected database that was accessible only to the IRB-approved study personnel. Because surveys were administered directly to students, demographic questions in the survey were limited to preserve anonymity.

### Statistical analysis

Quantitative data [including age, body mass index (BMI), NRS score, ODI score, and PSS-10 score] are presented as the means ± standard deviation. Qualitative data (including sex, major, and academic year) are presented as the frequencies.

Differences in quantitative data between the medical students and dental students were assessed using independent sample *t*-tests. Effect sizes were calculated using Cohen's d. Cohen's d effect size (small d = 0.2, medium d = 0.5, and large d = 0.8 effect sizes) were used to assess the significant effects (18). Differences in sex, ethnicity, smoking history, drinking history, and prevalence of MSP between the medical students and dental students were assessed using the chi-square test. Correlations of individual-level data were conducted with Spearman's correlation coefficient to examine the association. Effect sizes of 0.20 or lower were considered to demonstrate “small” relationships, while those ranging from 0.30 to 0.80 were considered to demonstrate “medium” relationships and those of 0.80 or greater “large” relationships (19). For multiple comparisons of prevalence among academic grades, the chi-square test followed by Bonferroni analyses were performed. Univariate and multivariate logistic regression analyses were performed to investigate factors influencing the prevalence of MSP in students. All variables found to have a statistically significant association (two-tailed, *P-value* < 0.05) in the univariate analyses were entered into multivariable logistic regression analyses *via* the forced-entry method (20). Odds ratios (ORs), 95% confidence intervals (CIs), and *P*-values were calculated for each independent variable. The model fit was assessed using the Hosmer–Lemeshow goodness of fit test (21). The Hosmer–Lemeshow statistic indicates a poor fit if the significance value is <0.05. All statistical analyses were performed using SPSS version 20.0 (IBM Corporation). The level of significance was set at *P* < 0.05.

## Results

### Demographic information

In total, 1,178 complete responses were received for a response rate of 79.6% (1,178/1,479), including 722 medical students and 456 dental students. The average age of the participants was 21.1 ± 1.7 years (range, 16–24), with 553 males and 625 females, and the average BMI was 21.1 ± 2.6. A summary of the characteristics of the participants is provided in Table 1.

**Table 1:** Demographic characteristics of included participants (*N* = 1,178).

	**Total**	**Clinical medicine**	**Stomatology**	**Cohen's d or Spearman's rho**	***P*-value**
Number of students	1,178	722	456	–	–
Academic year					
Grade 1	240	143	97	–	–
Grade 2	250	154	96	–	–
Grade 3	235	138	98	–	–
Grade 4	226	139	87	–	–
Grade 5	227	148	79	–	–
Age (years)	21.1 ± 1.7	21.2 ± 1.7	21.0 ± 1.7	0.12	0.12
BMI	21.1 ± 2.6	21.1 ± 2.6	21.1 ± 2.5	0	0.65
Gender				0.09	0.002
Male	553	365 (50.6%)	188 (41.3%)		
Female	625	357 (49.4%)	268 (58.7%)		
Ethnicity				0.006	0.83
Han	1,156	709 (98.2%)	447 (98.0%)		
Others	22	13 (1.8%)	9 (2.0%)		
Drinking				0.09^**^	0.001
Non-drinker (0 g/d)	981	620 (85.8%)	361 (79.1%)		
Mild drinker (0.1–9.9 g/d)	59	35 (4.8%)	24 (5.2%)		
Moderate drinker (10.0–29.9 g/d)	80	46 (6.3%)	34 (7.4%)		
Heavy drinker (30.0–49.9 g/d)	58	21 (2.9%)	37 (8.1%)		
Severe drinker (>50.0 g/d)	0	0 (0%)	0 (0%)		
Smoking				0.001	0.33
0 cigarette/d (non-smoker)	1,160	711 (98.5%)	449 (98.5%)		
0–5 cigarettes/d	11	8 (1.1%)	3 (0.7%)		
5–10 cigarettes/d	4	2 (0.3%)	2 (0.4%)		
10–15 cigarettes/d	2	0 (0%)	2 (0.4%)		
15–20 cigarettes/d	0	0 (0%)	0 (0%)		
>20 cigarettes/d	1	1 (0.1%)	0 (0%)		

** Correlation is significant at the 0.01 level (2-tailed).

As shown in Table 1, significantly more female students were seen in stomatology (58.7 vs. 49.4%, *P* = 0.002, Spearman's rho = 0.09) and had a higher proportion of alcohol consumption [20.8 vs. 14.1%, *P* = 0.001, Spearman's rho = 0.09, *P* < 0.01]. There were no significant differences in age, BMI, ethnicity, or smoking history between the students majoring in clinical medicine and stomatology.

### MSP in medical and dental students

#### NP

A total of 523 students reported NP (44.4%). Among them, 5.9% of the students had a history of neck trauma. Most students' pain occurred “sometimes” (73.0%), with an average NRS pain score of 2.9 ± 1.7 and a total ODI score of 18.8 ± 7.7. Among them, 299 medical students (41.4%) and 224 dental students (49.1%) had NP. There was a significant difference in the overall prevalence of NP between the two groups, with the prevalence of NP being significantly higher in dental students (*P* = 0.01, Spearman's rho = 0.08, *P* < 0.01). There was no significant difference between the two groups in trauma history, NRS score, frequency of pain or total ODI score (Table 2).

**Table 2:** Musculoskeletal pain in medical students and dental students.

	**Total**	**Clinical medicine**	**Stomatology**	**Cohen's d or Spearman's rho**	***P*-value**
Students number	1,178	722	456	–	–
Neck pain				0.08^**^	0.01
Yes	523 (44.4%)	299 (41.4%)	224 (49.1%)		
No	655 (55.6%)	423 (58.6%)	232 (50.9%)		
History of trauma			0.003	0.94
Yes	31 (5.9%)	18 (2.5%)	13 (2.9%)		
No	492 (94.1%)	281 (97.5%)	211 (97.1%)		
Frequency				−0.05	0.86
Almost never	63 (12.0%)	37 (12.3%)	26 (11.6%)		
Sometimes	382 (73.0%)	218 (72.9%)	164 (73.2%)		
Fairly often	70 (13.3%)	39 (13.0%)	31 (13.8%)		
Very often	8 (1.5%)	5 (1.6%)	3 (1.3%)		
NRS score	2.9 ± 1.7	3.0 ± 1.7	2.9 ± 1.6	0.06	0.79
ODI score	18.8 ± 7.7	18.5 ± 7.3	19.4 ± 8.3	0.12	0.05
Low back pain				0.07^*^	0.02
Yes	361 (30.6%)	203 (28.1%)	158 (34.6%)		
No	817 (69.4)	519 (71.9%)	298 (65.4%)		
History of trauma			−0.07	0.27
Yes	30 (8.3%)	14 (6.9%)	16 (10.1%)		
No	331 (91.7%)	189 (93.1%)	142 (89.9%)		
Frequency				−0.07	0.53
Almost never	55 (15.2%)	27 (13.3%)	28 (17.7%)		
Sometimes	253 (70.0%)	146 (71.9%)	107 (67.7%)		
Fairly often	45 (12.4%)	26 (12.8%)	19 (12.0%)		
Very often	8 (2.2%)	4 (1.9%)	4 (2.5%)		
NRS score	3.2 ± 1.8	3.4 ± 1.9	3.0 ± 1.7	0.22	0.04
ODI score	18.7 ± 7.9	18.4 ± 7.6	19.2 ± 8.4	0.10	0.11
Joint pain				0.10^**^	<0.001
Yes	182 (15.4%)	90 (12.5%)	92 (20.2%)		
No	996 (84.6%)	632 (87.5%)	364 (79.8%)		
History of trauma			0.16^*^	0.67
Yes	47 (25.8%)	22 (24.4%)	25 (27.2%)		
No	135 (74.2%)	68 (75.6%)	67 (72.8%)		
Frequency				0.11	0.89
Almost never	25 (13.7%)	13 (14.4%)	12 (13.0%)		
Sometimes	140 (76.9%)	69 (76.6%)	71 (77.1%)		
Fairly often	12 (6.6%)	5 (5.5%)	7 (7.6%)		
Very often	5 (2.7%)	3 (3.3%)	2 (2.1%)		
NRS score	3.2 ± 1.9	3.1 ± 1.9	3.2 ± 1.9	0.05	0.72
ODI score	17.7 ± 7.6	17.4 ± 7.1	18.3 ± 8.3	0.11	0.046

** Correlation is significant at the 0.01 level (2-tailed).

* Correlation is significant at the 0.05 level (2-tailed).

#### LBP

A total of 361 students reported LBP (30.6%), of which 8.3% had a history of trauma. Most of the students' pain occurred “sometimes” (70.0%), with an average NRS pain score of 3.2 ± 1.8 and a total ODI score of 18.7 ± 7.9. Among them, 203 medical students (28.1%) and 158 dental students (34.6%) reported LBP, and the prevalence of LBP was significantly higher in dental students (*P* = 0.02, Spearman's rho = 0.07, *P* < 0.05). However, the severity of LBP (presented as NRS score) was significantly higher in medical students (3.4 ± 1.9 vs. 3.0 ± 1.7, *P* = 0.04). There was no significant difference between the two groups in the history of trauma, frequency of pain, or total ODI score (Table 2).

#### JP

A total of 182 students reported joint pain (15.4%). The prevalence of each joint site was shoulder (17, 9.3%), elbow (6, 3.2%), wrist (10, 5.4%), hip (6, 3.2%), knee (122, 67.0%), ankle (17, 9.3%), and other joints (4, 2.1%). The knee is the most commonly affected region (data not shown in the table). A total of 25.8% of the students had a history of joint trauma. Most of the students' pain occurred “sometimes” (76.9%), with an average NRS pain score of 3.2 ± 1.9 and a total ODI score of 17.7 ± 7.6. Among them, there were 90 medical students (12.5%) and 92 dental students (20.2%). There was a significant difference in the overall prevalence of joint pain between medical students and dental students, and the prevalence was significantly higher in dental students (*P* < 0.001, Spearman's rho = 0.10, *P* < 0.01). It also had a greater impact on the daily life of dental students, with the ODI score being higher in this group (18.3 ± 8.3 vs. 17.4 ± 7.1, *P* = 0.046, Cohen's d = 0.11). There was no significant difference between the two groups in the history of trauma, the frequency of pain, or the total NRS score (Table 2).

### Co-occurrence of different types of MSP

Several students have suffered from the co-occurrence of NP, LBP, and JP. The results showed that 183 total students (15.5%) suffered from NP and LBP, 47 total students (4.0%) suffered from NP and JP, 25 total students (2.1%) suffered from LBP and JP, and 66 total students (5.6%) suffered from NP, LBP, and JP.

### Cross-grade differences of MSP

**Figure 1:**
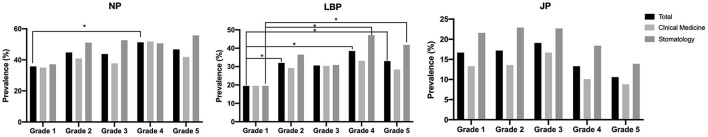
Cross-grade differences of musculoskeletal pain (*: significant difference).

Figure 1 shows the differences in the prevalence of NP, LBP, and JP among different grades. Statistical analysis showed that for total medical students, there was a difference in NP prevalence (*P* = 0.02) and LBP prevalence (*P* < 0.001) among academic grades. For dental students, there was also a difference in LBP prevalence (*P* = 0.001) among academic grades. However, the prevalence of JP, regardless of major, was not significantly different among grades (*P* = 0.08, *P* = 0.28, and *P* = 0.55 for total students, clinical medicine, and stomatology, respectively). Subgroup analysis showed that for total students, the prevalence of NP was significantly higher in Grade 4 than in grade 1, and the prevalence of LBP was significantly higher in Grade 2, Grade 4, and Grade 5 than in Grade 1. For dental students, it was significantly higher in Grade 4, and Grade 5 than in Grade 1. This data suggests that senior-year students tend to have a significantly higher prevalence of LBP than junior-year students.

### Risk factors for MSP in medical and dental students

To further explore the risk factors for MSP in medical and dental students, univariate and multivariate logistic regression analyses of multiple risk factors were performed. The relevant factors that may lead to the MSP of medical and dental students, including age, sex, BMI, academic year, major, ethnicity, drinking history, smoking history, PSS-10 score, exercise time, sleep time, study time, sit time, walk time, computer usage, and phone usage were included in the analysis.

Univariate regression analysis showed that age, sex, grade, major and PSS-10 score were all possible risk factors for NP. After multivariate regression analysis, sex (*P* < 0.001), major (*P* = 0.02), and PSS-10 score (*P* = 0.002) were risk factors for NP. Female students (OR = 1.552, 95% CI 1.221–1.973), major of stomatology (OR = 1.148, 95% CI 1.018–1.296), and high PSS-10 score (OR = 1.034, 95% CI 1.012–1.056) all contributed to NP (Table 3).

**Table 3:** Risk factors of musculoskeletal pain in medical and dental students.

	**Neck pain**			**Low back pain**			**Joint pain**		
	**Univariable analysis** **(*P*-value)**	**Multivariable analysis** **(*P-*value)**	**OR^a^** **(95% CI^b^)**	**Univariable analysis** **(*P-*value)**	**Multivariable analysis (*P-*value)**	**OR (95% CI)**	**Univariable analysis** **(*P-*value)**	**Multivariable analysis** **(*P-*value)**	**OR (95% CI)**
Age	0.01	0.62	1.045 (0.878–1.244)	0.10			0.03	0.09	0.922 (0.839–1.013)
Gender	<0.001	<0.001	1.552 (1.221–1.973)	<0.001	0.002	1.499 (1.154–1.946)	0.02	0.005	1.665 (1.169–2.373)
BMI	0.82			0.59			0.82		
Ethnicity	0.23			0.20			0.72		
Drinking	0.89			0.78			<0.001	<0.001	
Non-drinker									1
Mild drinker								0.01	2.303 (1.183–4.484)
Moderate drinker								<0.001	2.775 (1.576–4.885)
Heavy drinker								0.002	2.722 (1.452–5.100)
Severe drinker								N/A^C^	N/A
Smoking history	0.28			0.07			0.20		
PSS-10 score	<0.001	0.002	1.034 (1.012–1.056)	<0.001	<0.001	1.061 (1.034–1.089)	<0.001	<0.001	1.070 (1.033–1.108)
Major	0.009	0.02	1.148 (1.018–1.296)	0.02	0.04	1.143 (1.004–1.302)	<0.001	0.008	1.251 (1.061–1.475)
Grade	0.02	0.20		<0.001	<0.001		0.08		
1			1			1			
2		0.047	1.521 (1.005–2.303)		<0.001	2.180 (1.427–3.332)			
3		0.37	1.293 (0.734–2.279)		0.002	1.942 (1.263–2.985)			
4		0.15	1.673 (0.826–3.390)		<0.001	2.643 (1.730–4.039)			
5		0.47	1.370 (0.582–3.228)		<0.001	2.266 (1.472–3.489)			
Lifestyle									
Exercise	0.68			0.75			0.81		
Sleep	0.34			0.47			0.30		
Study	0.40			0.14			0.79		
Sit	0.23			0.19			0.41		
Walk	0.70			0.76			0.77		
Computer usage	0.08			0.32			0.32		
Phone usage	0.10			0.99			0.29		

a OR, odds ratio.

b CI,: Confidence interval.

c N/A, Not available.

For LBP, sex, grade, major and PSS-10 score were risk factors according to univariate regression analysis. Multivariate regression analysis showed that sex (*P* = 0.002), grade (*P* < 0.001), major (*P* = 0.04), and PSS-10 (*P* < 0.001) were all risk factors for LBP. Female sex (OR=1.499, 95% CI 1.154–1.946), major of stomatology (OR = 1.143, 95% CI 1.004–1.302), and high PSS-10 score (OR = 1.061, 95% CI 1.034–1.089) all contributed to LBP; senior grade also posed a risk factor for LBP (Table 3).

For JP, age, sex, major, drinking history, and PSS-10 score were all risk factors by univariate regression analysis. The results of multivariate regression analysis showed that sex (*P* = 0.005), major (*P* = 0.008), drinking history (*P* < 0.001), and PSS-10 score (*P* < 0.001) were risk factors. Students of female sex (OR = 1.665, 95% CI 1.169–2.373), majoring in stomatology (OR = 1.251, 95% CI 1.061–1.475), or with higher PSS-10 score (OR = 1.070, 95% CI 1.033–1.108) were more likely to have JP. Drinking was also significantly associated with a higher risk of JP (*P* < 0.001). Moderate and heavy drinkers were at higher risk of for JP (OR = 2.775, 95% CI 1.576–4.885, *P* < 0.001 and OR = 2.722, 95% CI 2.722 (1.452–5.100), *P* = 0.002, respectively) (Table 3).

### Factors influencing medical and dental students' intent to seek medical treatment for MSP

As shown in Table 4, when MSP occurred, the overall proportion of medical students seeking medical treatment was very low. Only 7 students with NP (1.3%), 12 students with LBP (3.3%), and 17 students with JP (9.3%) sought medical treatment, with the highest proportion seen in JP. To explore the influencing factors that may affect the intent to seek medical treatment, we also investigated the parents' education level, annual family income, and personal monthly income. The results showed that these factors had no statistical significance on the treatment intent of medical students. However, pain severity (NRS score) was significantly associated with students' intent regardless of pain sites (NP, LBP, or JP), and pain frequency also significantly affects students' intent who have JP. There were no associations between ODI scores and students' intent. This data suggests that students' intent to seek medical treatment is not affected by family education level or economic income level, but is affected by the pain severity.

**Table 4:** Medical and dental students' intent to seek medical treatment and possible influencing factors.

	**Neck pain**				**Low back pain**				**Joint pain**			
	**Seeking medical treatment**	**Conservative treatment**	**Spearman's rho**	***P*-value**	**Seeking medical treatment**	**Conservative treatment**	**Spearman's rho**	***P*-value**	**Seeking medical treatment**	**Conservative treatment**	**Spearman's rho**	***P*-value**
Mother's education			−0.02	0.53			0.02	0.78			0.08	0.30
University and above	3	105			2	68			3	45		
High school	1	103			4	79			3	31		
Junior high school	0	153			1	105			6	55		
Primary school and below	3	155			5	97			5	34		
Father's education			−0.06	0.19			−0.09	0.11			−0.009	0.80
University and above	3	140			5	91			5	59		
High school	3	120			4	80			6	41		
Junior high school	0	173			2	127			6	51		
Primary school and below	1	83			1	51			0	14		
Family annual income (in Chinese Yuan)			0.02	0.76			−0.01	0.77			−0.08	0.21
<49,999	2	115			3	66			5	20		
50,000–99,999	1	145			3	104			3	43		
100,000–199,999	1	130			3	98			5	56		
200,000–499,999	3	103			3	68			4	40		
500,000–999,999	0	17			0	12			0	5		
>1,000,000	0	6			0	1			0	1		
Personal monthly income (in Chinese Yuan)			0.02	0.78			0.04	0.59			−0.02	0.78
<1,000	2	197			3	137			6	58		
1,000–3,000	5	300			9	194			11	101		
>3,000	0	19			0	18			0	6		
Pain severity (NRS)	–	–	0.103^*^	0.02	–	–	0.17^**^	0.001	–	–	0.26^**^	0.001
Pain frequency			0.06	0.25			0.04	0.17			0.23^**^	0.002
Almost never	1	62			2	53			0	25		
Sometimes	3	379			7	246			12	128		
Fairly often	3	67			1	44			3	9		
Very often	0	8			2	6			2	3		
ODI score	–	–	−0.01	0.76	–	–	0.01	0.46	–	–	0.08	0.93

** Correlation is significant at the 0.01 level (2-tailed).

* Correlation is significant at the 0.05 level (2-tailed).

## Discussion

### The prevalence of MSP in Chinese medical and dental students is high

In this study, a cross-sectional survey was conducted among over 1,000 Chinese medical and dental students in a large medical college. The primary finding of this study is that the overall prevalence of MSP among Chinese medical students is very high. The prevalence of NP and LBP was 44.4 and 30.6%, respectively. Among these students, trauma is not the main cause of pain, as the rate of history of trauma in most students was low. The frequency of pain was moderate, with most students reporting pain occurring “sometimes.” The severity of pain (NRS score) was also moderate (with an average of ~3 points out of 10), and the ODI score, which assessed the severity of pain affecting daily life, was also moderate, fluctuating by ~18 points out of 90.

The reported prevalence of MSP in medical students is different across countries. However, all studies have demonstrated a relatively high prevalence of MSP. A study performed among Harvard medical students in the United States showed an overall prevalence of NP and LBP of 35% (74/210) and 47% (99/210), respectively (7). A Malaysian study on medical students showed that 45.7% had at least one site of MSP in the past week, and 65.1% had at least one site of MSP in the past year (9). An early cross-sectional study performed on Chinese medical students in 2005 surveyed 207 Chinese medical students and found that ~1/3 reported an ongoing MSP. The most commonly affected regions were the lower back (40.1%), neck (33.8%), and shoulders (21.7%). MSP significantly affected students' daily lives, with an average of 6.6 sick days taken from school and a higher probability of students suffering from mental pressure (6). Our study, with a significantly higher number of students, showed consistent results with this earlier study. Despite a similar trend, we demonstrated that the prevalence of NP was higher in modern medical students. On the one hand, limited physical exercise, increased duration of reading, and awkward neck posture are likely to be significantly associated with neck pain (22). On the other hand, the prevalence of smartphone addiction/overuse among medical students might be another important factor for this disparity (10, 23).

The current study also demonstrated that the prevalence of MSP (mainly NP and LBP) was significantly different among students' academic years. Subgroup analysis suggests senior-year students tend to have a significantly higher prevalence of LBP than junior-year students. This finding is not consistent with a survey of Harvard Medical students conducted by Jerry et al. (7). In their study, MSP did not differ significantly among medical students of different grades. In China's medical education system, there are 5 years of undergraduate education for medical and dental students. For senior year students (Year 4 and 5), they start internship and clinical work. Change of study mode and work mode might be an explanation for this disparity, but warrants further study to investigate.

### The prevalence of MSP is higher in dental students

Because of the different natures of the majors, students of different specialties may have different possibilities of experiencing MSP. Korean nurse students showed as high as 73.3% MSP in Smith's study (24), while Japanese nurse students showed 36.9% MSP (25). MSP was seen more commonly in nurses working in clinics, with a high number of 93.6% of nurses at a workplace in Korea (26) and 85.5% of nurses in Japan (27).

As such, our study included both medical students and dental students and compared the prevalence of MSP in these 2 groups. Another major finding of this study is that the prevalence of MSP in dental students was significantly higher than that of medical students, whether it was NP, LBP, or JP. However, the effect sizes were all <0.20, which should be considered as “small” according to Cohen's criteria on effect size. Moreover, the occurrence of joint pain significantly affected the daily life of dental students. This trend followed a study performed in the United Arab Emirates, which showed that the prevalence of MSP among dental students in at least one body site in the past week and the past year was 48.5 and 68.3%, respectively (13). Similar phenomena were seen in British dental students, as well. A study of British dental students revealed that 79% experienced pain, with 42% experiencing pain for 30 or more days in the past year, and lower back pain was most common (54%) and most frequently the worst area of pain (48%) (14). This may be due to the occupational nature of dental students, who often need to sit for a long time and maintain a fixed posture, especially after entering clinical work (28). Awkward postures that involve forward bending and repeated rotation of the head, neck, and trunk to one side are common occurrences during clinical work. As posture deviates more from neutral, the muscles that are responsible for the preferred side of rotating or bending become stronger, and the matching antagonistic muscles become elongated and weakened, creating a muscle imbalance (29).

### Risk factors for MSP in medical and dental students and their intent to seek medical treatment

The third finding of the study is that the MSP of medical and dental students is affected by many risk factors, including age, sex, major, academic year, and psychological conditions. Joint pain is even affected by drinking alcohol. Heavy drinkers are under a significantly higher risk for JP compared to non-drinkers (OR = 2.722). Harmful health behaviors, such as smoking and alcohol use, are frequent in the general population and may contribute to joint pain and inflammation (30), and gout in young adults is also becoming increasingly prevalent (31). The detailed mechanism of alcohol in inducing joint pain, however, warrants further research to elucidate. Our study also suggests that females and majoring in Stomatology are the risk factors for MSP. The OR value was all over 1.5 either for NP, LBP, or JP if female. In addition, PSS-10 is also a risk factor, which suggests for psychological problems, it is also necessary to intervene in time to avoid mental-induced physical problems. Notably, for LBP, students from Grade 5 also had a significantly higher risk compared to students from Grade 1 (OR = 2.266). However, our research has not found an impact of some recognized poor lifestyles on MSP, such as long-term learning and sitting. In addition, we also did not observe a significant association between the time spent on the computers or phones. However, the impact of the use of computers and phones on NP is close to statistical significance (Table 3). It is possible that the long-term use of computers and mobile phones still needs a certain cumulative effect on MSP. In the future, more studies with larger sample sizes and more rigorous and comprehensive designs are warranted to explore the possible causative factors that may contribute to MSP.

Our study also found that, when medical students have MSP, the overall intent to seek medical treatment is very low. Statistical analysis demonstrated that parents' education level, family income level, and personal income level have no significant impact on students' intention to seek medical treatment. Instead, pain severity is a key factor affecting students' intent. Nevertheless, the effect sizes were all small either for NP, LBP, or JP. One possible reason is that most medical students' pain, as presented by the NRS score, is relatively mild and may not have reached the point where the students need to see a doctor. However, a lack of medical education among students might be another possible reason. A similar situation was seen in prestigious colleges, such as Harvard University. A study revealed that Harvard medical students rated musculoskeletal education to be of major importance (3.8/5) but rated the amount of curriculum time spent on musculoskeletal medicine as poor (2.1/5) (32). The third-year students felt a low to an adequate level of confidence in performing a musculoskeletal physical examination and failed to demonstrate cognitive mastery in musculoskeletal medicine (32). Another curriculum analysis in Canada revealed that, on average, medical schools in Canada devoted only 2.26% of their curriculum time to musculoskeletal education (33). Our study reaffirms that medical school authorities should take measures to prevent MSP due to factors related to medical school. Careful attention from medical colleges is needed to increase students' awareness of this problem. Our study, therefore, contributes to a better understanding of MSP among medical and dental professionals.

### Limitations

This study has several limitations that need to be acknowledged. First, only undergraduate medical and dental students from one university in China were surveyed; thus, the results might not be generalizable to all medical and dental students in China. Despite these factors, the medical student samples were collected from a large public university that consists of students with diverse sociodemographic backgrounds and originated from various regions in China. Second, our study adopted a cross-sectional design, and we were therefore able to identify associations between exposure and outcomes but could not infer cause and effect. Third, in the questionnaire, we did not provide specific definitions of items such as the frequency of pain, which may make the questions unstraightforward to the participants. Information on whether the participants have underlying musculoskeletal diseases were also not acquired. Finally, the responses were based on self-report and may have been subject to recall bias, self-reporting bias, and a tendency to report socially desirable responses. In future work, more prospective studies with stringent designs are needed.

### Clinical significance and future research

MSPs are prevalent in working populations around the globe. Healthcare providers, because of the nature of their job duties, the incidence of MSP is significantly higher. MSP could significantly decrease work productivity, reduce the quality of life, cause chronic occupational disability, and constitute a major health challenge for healthcare providers (34). Our study re-affirmed this phenomenon and has demonstrated that MSP occurs even earlier, at the stage of medical university.

This study has several clinical significance. Firstly, medical students and healthcare providers should increase their own awareness of MSP. Whenever encountering situations including neck pain, back pain, or joint pain, medical students and healthcare providers should take regular check-ups and seek medical treatment when necessary. In daily work, it is recommended to take regular rest or change posture from time to time, to decrease the chance of MSP. Much attention should also be paid to decreasing the level of stress. Secondly, medical school or hospital authorities should take measures to prevent students or physicians from suffering from MSP. Curriculums of MSP should be provided to students and healthcare workers. Proposals of ergonomic training and education about MSP prevention can be considered. More importantly, the working environment should be improved to alleviate personnel's stress levels. The pace of work should be slowed down, and conflict resolution and supportive group systems are required to limit exposure to the factors associated with MSP (34). Lastly, the allocation of medical resources in China needs to be further optimized. Currently, in China, most patients still choose large tertiary hospitals for medical treatment, causing medical staff to work overload, significantly increasing their working hours and stress, and increasing the probability of suffering from MSP. How to improve the people's concept of seeking medical care and rationally distributing medical resources is what the Chinese government needs to continue to improve in the future.

In the future, more studies with stringent designs are still warranted. Firstly, more studies with larger sample sizes, with different races or ethnicities, or multi-center studies, are needed to eliminate the impact of individual differences between populations. Further prospective research can also be carried out to evaluate the outcome of MSP through dynamic observation at multiple time points. Secondly, the population of participants should be expanded. It should not only be limited to medical students, but also employees of large tertiary public hospitals, small public hospitals, and even private clinics. The specialty can also cover surgery, internal medicine, stomatology, and other subspecialties. Thirdly, objective data should also be obtained to evaluate MSP, rather than relying on the subjective feelings of the participants. For example, imaging examinations such as X-rays or MRI can be considered to further evaluate the existence and severity of MSP.

## Conclusions

The prevalence of MSP in Chinese medical and dental students is high, especially for NP and LBP, and is significantly higher in dental students than in medical students. The prevalence of NP and LBP were significantly different among academic grades. Female sex, PSS-10 score, and major of stomatology were risk factors for MSP. Students' intent to seek treatment for MSP was very low and was determined by the severity of pain. Careful attention from medical colleges is needed to increase students' awareness of this issue.

## Data availability statement

The original contributions presented in the study are included in the article/Supplementary material, further inquiries can be directed to the corresponding author/s.

## Ethics statement

The studies involving human participants were reviewed and approved by the First Affiliated Hospital of Fujian Medical University. The patients/participants provided their informed consent to participate in this study.

## Author contributions

YL and CZ conceived and designed the study. YL, XZ, HL, YH, and WZ collected the related clinical information, analyzed the data, and wrote the manuscript. YL, XZ, HL, WZ, and CZ analyzed the data. All authors contributed to the article and approved the submitted version.

## Funding

This work was supported by Joint Funds for the Innovation of Science and Technology, Fujian Province (2019Y9122), Foreign Cooperation Project of Science and Technology, Fujian Province (2020I0015), Startup Fund for Scientific Research of Fujian Medical University (2021QH1055), Talent Introduction Scientific Research Project of the First Affiliated Hospital of Fujian Medical University (YJRC3914), and Fujian Orthopaedic Bone and Joint Disease and Sports Rehabilitation Clinical Medical Research Center (2020Y2002).

## Conflict of interest

The authors declare that the research was conducted in the absence of any commercial or financial relationships that could be construed as a potential conflict of interest.

## Publisher's note

All claims expressed in this article are solely those of the authors and do not necessarily represent those of their affiliated organizations, or those of the publisher, the editors and the reviewers. Any product that may be evaluated in this article, or claim that may be made by its manufacturer, is not guaranteed or endorsed by the publisher.
