# Supplementary material for: Musculoskeletal pain is prevalent in Chinese medical and dental students: A cross-sectional study
Source: Front Public Health. 2022 Nov 24;10:1046466. doi: 10.3389/fpubh.2022.1046466 (PMC9730812; doi:10.3389/fpubh.2022.1046466)
Supplement: Supplementary file 1 [file Table_1.docx]

**Appendix. Survey questionnaire**

**Section A Demographic information**

A.1 Age ___ y.o.

A.2 Gender

[ ] Male

[ ] Female

A.3 Height ___ cm

A.4 Weight___ kg

A.5 major

[ ] Clinical Medicine

[ ] Stomatology

A.6 Academic grade

[ ] Grade 1

[ ] Grade 2

[ ] Grade 3

[ ] Grade 4

[ ] Grade 5

A.7 Ethinicity

[ ] Han

[ ] Others

A.8 History of hypertension, diabetes, or other congenital diseases

[ ] Yes

[ ] No

A.9 Drinking alcohol (daily consumption of ethanol, in past six months. One unit of beer, wine and liquor on average contain 30, 90 and 200 g of ethanol, respectively.)

[ ] non-drinker (0 g/d)

[ ] daily consumption (0.1–9.9 g)

[ ] daily consumption (10.0–29.9 g/d)

[ ] daily consumption (30.0–49.9 g/d)

[ ] daily consumption (> 50.0 g/d)

A.10 Smoking (daily consumption of cigarettes, in past six months)

[ ] 0 cigarette/d (nonsmoker)

[ ] 0-5 cigarettes/d

[ ] 5-10 cigarettes/d

[ ] 10-15 cigarettes/d

[ ] 15-20 cigarettes/d

[ ] >20 cigarettes/d

A.11 Mother’s education level

[ ] University and above

[ ] High school

[ ] Junior high school

[ ] Primary school and below

A.12 Father’s education level

[ ] University and above

[ ] High school

[ ] Junior high school

[ ] Primary school and below

A.13 Family annual income

(in Chinese Yuan)

[ ] < 49,999

[ ] 50,000–99,999

[ ] 100,000–199,999

[ ] 200,000–499,999

[ ] 500,000–999,999

[ ] > 1,000,000

A.14 Personal monthly income (in Chinese Yuan)

[ ] <1,000

[ ] 1,000-3,000

[ ] >3,000

**Section B Musculoskeletal pain**

B.1. In a typical day, how much time do you spent on

Exercising: _____hours

Sleeping: ____hours

Studying: ____hours

Sitting: ____hours

Walking: ____hours

Computer usage: ____hours

Phone usage: ____hours

B.2. If you have a neck pain?

[ ] Yes

[ ] No

B.3. Is there a history of neck trauma?

[ ] Yes

[ ] No

B.4. Please mark the severity of your pain (0 for ‘no pain’ to 10 for ‘most severe pain’).

No pain Most severe pain

0 1 2 3 4 5 6 7 8 9 10


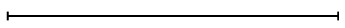


B.5. How frequently does the pain manifest?

[ ] Almost never

[ ] Sometimes

[ ] Fairly often

[ ] Very often

B.6. What you do for your neck pain?

[ ] Seeking medical treatment (consult a doctor)

[ ] Conservative treatment (rest, wearing braces or protective equipment, or taking analgesic drugs)

B.7. If you have low back pain?

[ ] Yes

[ ] No

B.8. Is there a history of lumbar trauma?

[ ] Yes

[ ] No

B.9. Please mark the severity of your pain (0 for ‘no pain’ to 10 for ‘most severe pain’).

No pain Most severe pain

0 1 2 3 4 5 6 7 8 9 10


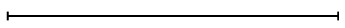


B.10. How frequently does the pain manifest?

[ ] Almost never

[ ] Sometimes

[ ] Fairly often

[ ] Very often

B.11 What you do for your back pain?

[ ] Seeking medical treatment (consult a doctor)

[ ] Conservative treatment (rest, wearing braces or protective equipment, or taking analgesic drugs)

B.12. If you have a joint pain?

[ ] Yes

[ ] No

B.13. Joint pain site

[ ] Shoulder

[ ] Elbow

[ ] Wrist

[ ] Hip

[ ] Knee

[ ] Ankle

[ ] other joints: _______

B.14.Whether the corresponding joint has a history of trauma?

[ ] Yes

[ ] No

B.15. Please mark the severity of your pain (0 for ‘no pain’ to 10 for ‘most severe pain’).

No pain Most severe pain

0 1 2 3 4 5 6 7 8 9 10


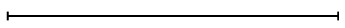


B.16. How frequently does the pain manifest?

[ ] Almost never

[ ] Sometimes

[ ] Fairly often

[ ] Very often

B.17. What you do for your joint pain?

[ ] Seeking medical treatment (consult a doctor)

[ ] Conservative treatment (rest, wearing braces or protective equipment, or taking analgesic drugs)

**Section C Impact of musculoskeletal pain on your life (PSS-10)**

The following 10 questions ask you about your feelings and thoughts during the last month. Please mark how often you felt or thought a certain way. (1=Never, 2=Almost Never, 3=Sometimes, 4=Fairly Often, 5=Very Often)

C.1. In the last month, how often have you been upset because of something that happened unexpectedly? _______

C.2. In the last month, how often have you felt that you were unable to control the important things in your life? _______

C.3. In the last month, how often have you felt nervous and stressed? _______

C.4. In the last month, how often have you felt confident about your ability to handle your personal problems?_____

C.5. In the last month, how often have you felt that things were going your way ?_____

C.6. In the last month, how often were you unable to cope with all the things that you had to do?_____

C.7.In the last month, how often have you been able to control irritations in your life?_____

C.8. In the last month, how often have you felt that you were on top of things? _____

C.9. In the last month, how often have you been angered because of things that were outside of your control? _____

C.10. In the last month, how often have you felt difficulties were piling up so high that you could not overcome them? _____

**Section D Impact of musculoskeletal pain on your life (ODI)**

D.1. Pain Intensity:

[ ] I have no pain at the moment.

[ ] The pain is very mild at the moment.

[ ] The pain is moderate at the moment.

[ ] The pain is fairly severe at the moment.

[ ] The pain is very severe at the moment.

[ ] The pain is the worst imaginable at the moment.

D.2. Personal Care (washing, dressing, etc.):

[ ] I can look after myself normally without causing extra pain.

[ ] I can look after myself normally, but it causes a little extra pain.

[ ] It is painful to look after myself, and I am slow and careful.

[ ] I need some help, but manage most of my personal care.

[ ] I need help every day in most aspects of my care.

[ ] I do not get dressed, wash with difficulty and stay in bed.

D.3. Lifting:

[ ] I can lift heavy weights without increased pain.

[ ] I can lift heavy weights, but it causes increased pain.

[ ] Pain prevents me from lifting heavy weights off the floor, but I can manage if the weights are conveniently positioned (ex. on a table).

[ ] Pain prevents me from lifting heavy weights, but l can manage light to medium weights if they are conveniently positioned.

[ ] I can lift only very light weights.

[ ] I cannot lift or carry anything at all.

D.4. Sleeping:

[ ] I have no trouble sleeping.

[ ] My sleep is slightly disturbed (less than 1 hoursleepless).

[ ] My sleep is mildly disturbed (1-2 hours sleepless).

[ ] My sleep is moderately disturbed (2-3 hours sleepless).

[ ] My sleep is greatly disturbed (3-5 hours sleepless).

[ ] My sleep is greatly disturbed (5-7 hours sleepless).

D.5. Sitting:

[ ] I can sit in any chair as long as I like

[ ] I can only sit in my favorite chair as long as I like

[ ] Pain prevents me sitting more than one hour

[ ] Pain prevents me from sitting more than 30 minutes

[ ] Pain prevents me from sitting more than 10 minutes

[ ] Pain prevents me from sitting at all

D.6. Standing:

[ ] I can stand as long as I want without extra pain

[ ] I can stand as long as I want but it gives me extra pain

[ ] Pain prevents me from standing for more than 1 hour

[ ] Pain prevents me from standing for more than 10minutes

[ ] Pain prevents me from standing for more than 3minutes

[ ] Pain prevents me from standing at all

D.7. Walking:

[ ] Pain does not prevent me walking any distance

[ ] Pain prevents me from walking more than 2 kilometers

[ ] Pain prevents me from walking more than 1 kilometer

[ ] Pain prevents me from walking more than 500 meters

[ ] I can only walk using a stick or crutches

[ ] I am in bed most of the time

D.8. Social life:

[ ] My social life is normal and gives me no extra pain

[ ] My social life is normal but increases the degree of pain

[ ] Pain has no significant effect on my social life apart from limiting my more energetic interests,e.g sports

[ ] Pain has restricted my social life and I do not go out as often

[ ] Pain has restricted my social life to my home

[ ] I have no social life because of pain

D.9. Traveling:

[ ] I can travel anywhere without pain

[ ] I can travel anywhere but it gives me extra pain

[ ] Pain is bad but I manage journeys over two hours

[ ] Pain restricts me to journeys of less than one hour

[ ] Pain restricts me to short necessary journeys under 30minutes

[ ] Pain prevents me from travelling except to receive treatment

D.10. Reading:

[ ] I can read as much as I want to with no pain.

[ ] I can read as much as I want to with slight pain.

[ ] I can read as much as I want to with moderate pain.

[ ] I cannot read as much as I want because of moderate pain.

[ ] I cannot read as much as I want because of severe pain.

[ ] I cannot read at all.

D.11. Headaches:

[ ] I have no headaches at all.

[ ] I have slight headaches which come infrequently.

[ ] I have moderate headaches which come infrequently.

[ ] I have moderate headaches which come frequently.

[ ] I have severe headaches which come frequently.

[ ] I have headaches almost all the time.

D.12. Concentration:

[ ] I can concentrate fully when I want to with no difficulty.

[ ] I can concentrate fully when I want to with slight difficulty.

[ ] I have a fair degree of difficulty in concentrating when l want to.

[ ] I have a lot of difficulty in concentrating when I want to.

[ ] I have a great deal of difficulty in concentrating when l want to.

[ ] I cannot concentrate at all.

D.13. Work:

[ ] I can do as much work as I want to.

[ ] I can only do my usual work, but no more.

[ ] I can do most of my usual work, but no more.

[ ] I cannot do my usual work.

[ ] I can hardly do any work at all.

[ ] I cannot do any work at all.

D.14. Driving:

[ ] I can drive my car without any pain.

[ ] I can drive as long as I want with slight pain.

[ ] I can drive as long as I want with moderate pain.

[ ] I cannot drive as long as I want because of moderate pain.

[ ] I can hardly drive at all because of severe pain.

[ ] I cannot drive my car at all.

D.15. Recreation:

[ ] I am able to engage in all of my recreational activities with no pain.

[ ] I am able to engage in all of my recreational activities with some pain.

[ ] I am able to engage in most, but not all of my recreational activities because of pain.

[ ] I am able to engage in a few of my recreational activities because of pain.

[ ] I can hardly do any recreational activities because of pain.

[ ] I cannot do any recreational activities at all.
